# Comparison of Antibiotic Use and the Frequency of Diseases Depending on the Size of Herd and the Type of Cattle Breeding

**DOI:** 10.3390/ani14131889

**Published:** 2024-06-27

**Authors:** Robert Kupczyński, Michał Bednarski, Marcin Sokołowski, Wojciech Kowalkowski, Katarzyna Pacyga

**Affiliations:** 1 Department of Environment Hygiene and Animal Welfare, The Faculty of Biology and Animal Science, Wroclaw University of Environmental and Life Sciences, 38c Chelmonskiego St., 50-375 Wroclaw, Poland; katarzyna.pacyga@upwr.edu.pl; 2 Department of Epizootiology and Clinic of Bird and Exotic Animals, Faculty of Veterinary Medicine, Wroclaw University of Environmental and Life Sciences, 47 Grunwaldzki Sq., 50-366 Wroclaw, Poland; michal.bednarski@upwr.edu.pl; 3 OSI Food Solutions, 14-100 Ostróda, Polandwkowalkowski@osieurope.com (W.K.)

**Keywords:** antimicrobials, antibiotic consumption monitoring, treatment incidence, dairy cattle

## Abstract

**Simple Summary:**

Various substances are used to treat animal diseases, including antimicrobials (such as antibiotics, antivirals, antifungals, and antiparasitics). The use of antibiotics to combat bacterial infections is beneficial in stopping the spread of disease and protecting the rest of the herd, thus promoting animal health and welfare and contributing to food safety. Reducing antimicrobial use is one of the key ways to decrease the occurrence of antimicrobial resistance. Monitoring and overseeing the use of antibiotics in livestock helps reduce their application. Our research focused on comparing antibiotic use and the frequency of diseases in three types of cattle farming in Poland (medium dairy farms, large dairy farms, and large beef farms).

**Abstract:**

Diseases are responsible for losses in livestock production by increasing animal mortality and reducing productivity. The administration of antibiotics can help mitigate these negative effects. However, inappropriate use can lead to severe complications, such as raising antibiotic resistance. The purpose of this study was to perform a comparative analysis of antibiotic use and disease frequency over four years, based on the size of dairy farms and the type of farm. The study covered a 4-year period and included medium dairy farms (20–50 cows, n = 13), large dairy farms (>250 cows, n = 8), and large beef farms (n = 8). The collected data involved antimicrobial use but also included farm demographics, animal health, disease frequency, and herd management practices. The criteria used to categorise antibiotics into groups A–D were based on the EMA guidelines. The carried-out study showed that the large dairy cattle farms had the highest antibiotic consumption (18.29 mg·PCU^−1^), due to the high frequency of diseases, and consequently, the treatment of calf (diarrhoea, lung inflammations) and cow diseases (general treatment and mastitis). Cattle on large beef farms suffer mainly from general diseases caused by maintenance and herd management conditions. The use of restrict antibiotics was, in some cases, unjustified (antibiotics for dry cow therapy). Future studies should consider a larger number of farms, taking into account the given direction of cattle production.

## 1. Introduction

Farmers, ranchers, and veterinarians prioritise raising healthy and safe animals for food production. However, despite providing adequate nutrition, maintaining a hygienic environment with good air quality, vaccinating animals, and implementing biosecurity protocols, diseases are still observed among dairy cattle. To prevent sick animals from suffering, it is crucial not to avoid or delay their treatment [1,2,3]. Various substances are used to treat animal diseases, including antimicrobials (such as antibiotics, antivirals, antifungals, and antiparasitics) [2]. The use of antibiotics to combat bacterial infections is beneficial in terms of stopping the spread of disease and protecting the rest of the herd, thus promoting animal health and welfare, and contributing to food safety [1]. Furthermore, in livestock systems, antibiotics have also found use as prophylaxis and metaphylaxis, which is now being limited [1,3,4]. They are widely used to minimise foodborne pathogens and prevent foodborne diseases, which are responsible for high morbidity and mortality worldwide [3,5]. In Europe, the European Medicines Agency (EMA) has been monitoring sales of veterinary antimicrobials since 2009 to promote their prudent use in animals through the European Surveillance of Veterinary Antimicrobial Consumption (ESVAC). Total antibiotic sales declined by 53% between 2011 and 2022, reaching the lowest level ever recorded [6].

Antimicrobial resistance (AMR) occurs when bacteria, viruses, fungi, and parasites no longer respond to antimicrobial drugs. As a result of drug resistance, antibiotics and other antimicrobial drugs become ineffective, making infections difficult or impossible to treat, thereby increasing the risk of spread of the disease and posing a serious threat to human health [7,8,9]. The problem of antibiotic resistance has gained significant importance in recent years, and infections caused by multi-resistant bacteria are expanding rapidly and are extremely detrimental to human health [10,11]. There are statistical data that, based on predictive models, estimated that 4.95 million deaths in the world were associated with bacterial AMR in 2019, including 1.27 million deaths directly attributable to bacterial AMR [9]. According to the cited data, six common pathogens in humans and animals are responsible for the most significant cases of resistance or multi-resistance (*Escherichia coli*, *Staphylococcus aureus*, *Klebsiella pneumoniae*, *Streptococcus pneumoniae*, *Acinetobacter baumannii*, and *Pseudomonas aeruginosa*) [9].

A variety of organisms from non-human sources (animals, water, food, or the environment) may also transmit resistance determinants to human pathogens [12]. The continued use of antibiotics at the sub-therapeutic level poses a serious threat to human health due to the development of resistant microorganisms and through the entry of residues into the food supply chain and the environment [1]. The use of antimicrobials in livestock production has been recognised as one of the main causes of antibiotic resistance in human and animal populations [5,10]. Antimicrobial stewardship (AMS) programmes in animal production systems and human health are essential to combat antimicrobial resistance [4]. Furthermore, several global actions aimed at the prudent use of antibiotics and the reduction of antibiotic resistance have been proposed by organisations such as the World Organization for Animal Health (WOAH), the Food and Agriculture Organization of the United Nations (FAO), and the World Health Organization (WHO). These joint efforts have been guided by the concept of “One Health” for more than a decade, which considers health in the context of human, animal, and environmental relationships [13]. The effects of drugs and their metabolites impact not only microorganisms but also invertebrates, aquatic environments, and soil [14,15]. Due to the advancing antibiotic resistance, EU countries have introduced legislation not only regarding the principles of monitoring antibiotic usage but also their prudent use [16].

Reducing the development and spread of antimicrobial resistance is crucial to: (1) maintain the world’s ability to cure diseases in humans, animals, and plants; (2) diminish food safety and security risks; (3) protect the environment; and (4) support progress toward the Sustainable Development Goals [5,17]. However, such a holistic approach requires knowledge about the causes of animal diseases and the scale of the problem, which can be determined by monitoring antibiotic usage. The type of disease and its prevalence determine the need for antibiotic therapy. The purpose of the study was to conduct a comparative analysis of antibiotic usage and the frequency of diseases over a four-year period, based on the size of dairy farm (medium vs. large) and the type of production (meat farms). We hypothesise that the type and size of the farm influence antibiotic usage, although other factors not related to the size of the farm or maintenance conditions may also play a role.

## 2. Materials and Methods

### 2.1. Study Design and Farm Data

The study population consisted of medium dairy farms (20–50 cows, n = 13) which constituted family farms, large dairy farms (>250 cows, n = 8), and large beef farms (n = 8). The selection of farms was purposeful, and it was based on varying herd size, with an additional criterion being the agreement to share all necessary data. The farms for the study were randomly selected from three regions in Poland (northeast, central, and southwest), and a cross-sectional survey was conducted through in-person interviews to complete a questionnaire and retrospectively collect on-farm medicine records. The study covered a full four years (2018–2022). All herds used in the study were registered, and owners gave their informed consent to participate in the survey and to provide data on animal treatment (from electronic systems or animal treatment cards). Farm visits were conducted quarterly, always by the same person. The collected data included antimicrobial usage, farm demographics, animal health and disease frequency, and herd management practices. Data were recorded based on the best knowledge of the farmer and farm documentation. Data were collected into a spreadsheet, the scope of which is included in Supplementary Materials: data sheet template.

### 2.2. Antimicrobial Consumption Data

The researcher visiting the farm filled out a questionnaire on the use of antibiotics to treat various diseases (recording the use of antibiotics overall as general treatment), treating mastitis during lactation (LC), and the use of antibiotics for dry cow therapy (DC). To track antibiotic usage, a spreadsheet was developed, where data from each farm were entered for each quarter of the year. At the same time, the frequency of specific diseases was recorded based on the size of the relevant group (e.g., calves, dairy cows, beef cattle). Morbidity analysis included only those cases in which antibiotics were used during treatment. In assessing antibiotic usage, the following factors were considered: frequency of administration to animals, category according to EMA [18], type of antibiotics (restrict or others), and the level of risk of developing antimicrobial resistance [18]. The criteria for categorising antibiotics into categories A–D according to EMA [18] are described below:

Category A (“Avoid”) includes antibiotic classes not authorised for veterinary use but authorised for human use in the EU. Medications from this group are not used in animals in Poland.

Category B (“Restrict”) includes substances listed as the highest priority Critically Important Antimicrobials (HPCIAs) by the WHO, except for macrolides and those classes included in Category A. This category contains quinolones, 3rd- and 4th-generation cephalosporins, and polymyxins.

While there are generally alternatives for these substances in human medicine in the EU, there are fewer alternatives in veterinary medicine for certain indications.

Category D (“Prudence”) is the lowest risk category. Although the risk to public health from the veterinary use of substances in this category is considered low, several substances in this category are listed as WHO CIAs (aminopenicillins, natural penicillins, and isoxazolylpenicillin). Calculations of antibiotic amounts (categories A–D), in total milligrammes of active ingredient per population correction unit (PCU), were carried out according to the following formula:

$$Antibiotic\;usage\;(mg\cdot {PCU}^{-1})=\frac{Active\;substanceinmg\;administered\;(mg)}{Number\;of\;animals\times standard\;weight(kg)}$$

Antimicrobials were grouped by antimicrobial class (e.g., penicillin, fluoroquinolone, quinolones, and third- and fourth-generation cephalosporins) and according to the route of administration (e.g., injectable, intramammary). A separate spreadsheet was created in which, based on the trade names of the drugs, units of medication were defined in millilitres for injectable drugs, the number of boluses or tablets, grammes for powders, and the number of tubes for the treatment of mastitis. In the next step, the quantity of each drug was converted into the amount of active ingredient.

### 2.3. Statistical Analysis

The farms studied only partially reflect the issue of antibiotic usage throughout the entire population; therefore, the incidence was treated descriptively. For data characterising antibiotic usage in mg·PCU^−1^, commercially available statistical software, Statistica 13^®^, was used. Descriptive statistics were analysed in terms of mean value, standard deviation (SD), and standard error of mean (SEM) for normally distributed variables. Baseline variable analyses were performed across different types of farms (medium dairy farms, large dairy farms, and beef farms) using analysis of variance (ANOVA). Differences between means were analysed for significance (*p* < 0.05) using the Tukey post hoc test.

## 3. Results

Data on the size of monitored herds varied significantly due to the type of cattle production and the livestock production profile (Table 1). All large dairy herds, in addition to cows and calves, also raised heifers intended for breeding. In dairy herds, calves aged 1 to 8 weeks were sold to other farms (generally at a young age). Among the beef herds studied, two did not maintain cows; instead, the operation was based on the purchase of young animals. Due to the distribution of the herds, there was no connection between the dairy herds and the beef herds. The use of antibiotics for dry cow therapy was more common in large dairy herds compared to medium farms. The determination of the type of bacteria that cause infections and antibiotic resistance before starting treatment was at a very low level, occurring mainly before starting LC therapy.

In large dairy herds, the incidence of mastitis was significantly higher than in medium (family) herds (15.85 vs. 5.98). Most medium dairy herds grazed their animals on pastures, while large dairy farms kept cows in free-stall barns (three farms provided cows with access to outdoor runs). As a result, in large dairy herds, the incidence of laminitis was 7.57 (ranging from 4.70 to 14.43). Gastrointestinal disorders in calves had the highest frequency in large dairy farms, whereas it was significantly lower in medium dairy farms and beef herds. Similar patterns were observed for respiratory diseases. Conditions listed as “other” were quite varied, and Table 1 includes only those “other” conditions that required antibiotic treatment. Metabolic disorders were not included in these data.

Table 2 shows the average antibiotic usage over a 4-year period between individual farms. The highest (*p* < 0.01) annual antibiotic usage was found in large dairy farms (mean 18.29), while the lowest usage was recorded in medium dairy farms and beef herds. In all types of herds, the largest (*p* < 0.01) quantities of antibiotics in mg·PCU^−1^ were used to treat general diseases. Restrict antibiotics (3 = third- and fourth-generation cephalosporins, polymyxins—colistin and polymyxin B, and fluoroquinolones) were used in the largest amounts (*p* < 0.01) in large dairy herds. The quantity of restrict antibiotics used there was about 5 times greater than in beef herds or medium dairy farms, with most of their use related to general treatment (Table 2). In large dairy herds, the most significant amounts (*p* < 0.05) of category D antibiotics were used to treat mastitis during lactation, while in medium dairy farms, category D antibiotics were predominant for this treatment. Statistically significant differences (*p* < 0.01) of restrict antibiotics were used for dry cow therapy more in large dairy farms compared to medium dairy farms. In dairy farms, 100% of antibiotic use was for general treatment.

**Figure 1:**
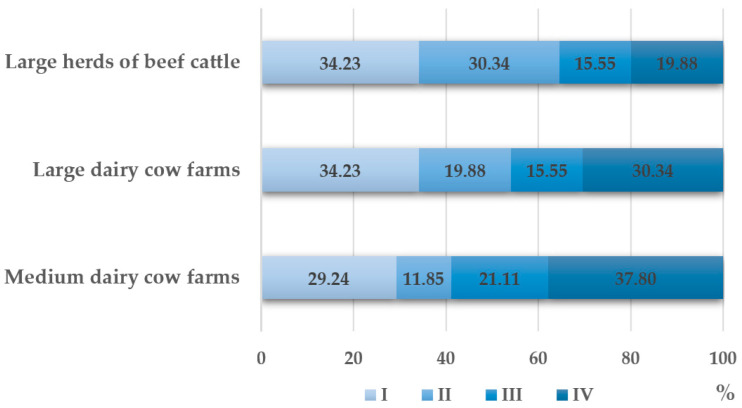
Antibiotic usage in individual farms with a focus on each quarter of the year (I–IV).

Overall antibiotic usage in individual quarters of the year varied significantly (Figure 1). In large dairy and beef herds, the highest usage was recorded in the first quarter of the year, while in medium dairy farms, it was in the fourth quarter. During these periods, all cattle production and age groups were at their highest.

## 4. Discussion

During the past 50 years, world meat production has increased rapidly, from 70.57 mln tonnes in 1961 to 355.46 mln tonnes in 2022; hereby, the total production of beef meat has doubled (from 28.75 to 76.25 mln tonnes, respectively) [19]. On the other hand, milk per animal increased from 884.50 kg in 1961 to 1360.40 kg in 2022 [20], while only in the European Union milk production (mainly from cows) amounted to 160.1 million tonnes [21]. It is estimated that in the EU alone, there are 23.5 million dairy cows, and each cow produces 25 kg of milk per day [21]. Implementing changes in breeding practices has led to higher growth rates and better feed conversion efficiency of heifer and, steers [22]. However, it is of great importance to provide antibiotic-free animal products [9]. Simultaneous and combined use of antibiotics in food-producing animals and humans has been proven to have a positive effect on the occurrence of resistance in both populations and, consequently, poses a serious threat to human life [23] and the long-term sustainability of the animal industry. Monitoring global AMU is crucial for tracking progress in addressing the causes of antimicrobial resistance [24]. Therefore, consumers and other stakeholders are paying greater attention to the consumption of antibiotics in food-producing animals. The best way to improve the sustainability of antimicrobials use without contributing to animal welfare issues is to identify preventive approaches and management practices to reduce the morbidity of food-producing animals [25]. In the study, data from private monitoring were used, which was carried out jointly with the food production sector. National milk production is characterised by large regional variations. According to Statistics Poland [26], in 2023, up to 64% of Poland’s dairy herds consisted of up to 9 cows, but 59% of the population under dairy control was kept in stables with more than 50 heads [27]. Among the herds included in the dairy performance audit, herds with a size between 20 and 49 cows represented 35.4%. Within this range were the self-monitored herds identified as medium. In 2023, it is estimated that the average productivity of dairy cows on farms not under evaluation of dairy performance was 6601 kg of milk per head, while those under control were 9150 kg [26,27]. The large herds included in the study had a higher yield (average of 11,455 kg of milk per year).

High morbidity due to infectious diseases and the increased use of antibiotics have become a major challenge in the beef production sector [24]. In the work of Rahman and Hollis [23], the average antibiotic usage by class for food-producing animals was presented. The class of tetracyclines (107.38 t) and penicillins (73.04 t) were the most used antibiotics, followed by sulphonamides (32.34 t), macrolides (21.70 t), polymyxins (16.19 t), aminoglycosides (12.46 t), fluoroquinolones (8.97 t), cephalosporins (5.77 t), trimethoprim (4.77 t), and amphenicols (3.68 t); carbapenems were not included in their comparison [23]. In the dairy industry, mastitis is one of the most common inflammations and affects 40% of cattle in herds. This disease represents a major economic burden, with more than EUR 185 per cow annually in the EU and USD 35 billion worldwide. These financial losses can be assigned to a wide range of factors, such as reduction in milk quality and production, discarded milk, veterinary service fees, drug treatment costs, and premature slaughter [22,28]. The demonstrated correlations, especially considering the use of restrict antibiotics, indicate the potential for reducing their use (e.g., dairy cow therapy).

The frequency of mastitis observed in the study was highest in large farms. Furthermore, on these farms, in the first and second quarters of the year, the prevalence significantly exceeded that of the other quarters. This was reflected in the use of antibiotics. However, in medium dairy farms, the low frequency of mastitis at 5.98% was significantly variable between farm averages, with a range from 0.29% to 27.20%. In large dairy farms, with an average mastitis frequency of 15.85%, the frequency range between farms was also quite large. Infectious mastitis can be caused by *Staphylococcus aureus*, *Streptococcus agalactiae*, and *Mycoplasma bovis* [28]. For example, antibiotic treatment of *Staphylococcus aureus*, an important causative agent of mastitis in cattle, includes cefoperazone, ceftiofur, enrofloxacin/ciprofloxacin, erythromycin, methicillin, neomycin, penicillin, penicillin–novobiocin, pirlimycin, and sulphonamide–trimethoprim [29]. In turn, mammary infections (called environmental mastitis) can be the result of infection by *Escherichia coli*, *Klebsiella pneumoniae*, and coagulase-negative staphylococci (CNS) [28]. However, considering the increasing resistance of bovine mastitis bacteria strains, namely *S. aureus* to penicillin (25% in Europe, 66% in China), CNS to penicillin (29.1% in Europe, 62% in China), CNS to oxacillin (56.4% in Europe, 84% in China), *E. coli* to tetracycline (14.5% in Europe, 10% in China), *Klebsiella* spp. to tetracycline (19.5% in Europe, 32% in China), worldwide attention should be paid to the search for alternative treatments for bacterial diseases [28]. The aforementioned *E. coli* can cause the onset of various infections, but it most notably contributes to the development of mastitis in adult dairy cows, yet also intestinal or septicaemic infections in calves. To a lesser extent, this type of bacteria triggers the formation of cystitis, peritonitis, metritis, sepsis-derived meningitis, and wound infections. Treatment of these conditions involves a wide range of antibiotics, including ampicillin/amoxicillin, amoxicillin–clavulanic acid, apramycin, colistin, enrofloxacin/ciprofloxacin, gentamicin, neomycin, paromomycin, sulphonamide–trimethoprim, tetracyclines, and 3GC [29]. It is estimated that about 80% of antibiotics administered in the dairy industry are targeted for the control and treatment of mastitis [22]. In our study, in dairy herds (regardless of their size), the highest amounts of antibiotics, expressed in mg·PCU^−^^1^, were used in general treatment. This type of treatment dominated in meat herds. 

The morbidity rates used in this study were cases under antibiotic treatment. Mild illnesses, where antibiotics were not used, were not included in the statistics. Lameness is the second condition that has an adverse impact on herd productivity, and the third most common cause of culling or premature elimination from the herd. It also negatively affects animal welfare and production economics [30,31]. It refers to any foot or leg condition of infectious or non-infectious origin that negatively affects the mobility, posture, and gait of the cow [30]. Cows with severe lameness suffer due to their inability or reluctance to stand or walk on the affected legs. Loss of weight and weakness can lead to complete inability to walk [32]. Causes can be non-infectious (e.g., white line disease, sole ulcer, sole haemorrhage, interdigital hyperplasia) or infectious (e.g., digital dermatitis, interdigital dermatitis, interdigital phlegmon [30,32]). Digital dermatitis (DD) is highly contagious, is transmitted throughout the dairy herd, and persists chronically. Species that are isolated in cases of DD include: *Treponema*, *Bacteroides*, *Mycoplasma* spp., *Campylobacter* spp., and *Amoebophilus asiaticus* [30]. The consequences of this disease include reduced milk production (approximately 20%) and reproductive performance, treatment costs, and increased risk of death and culling [31]. The estimated cost of clinical lameness in dairy cattle is approaching USD 500 per case [32]. Most cases of lameness can be successfully managed with prompt and effective therapeutic claw trimming, surgery, analgesia with the use of non-steroidal anti-inflammatory drugs, as well as the application of weight-bearing modifying blocks [31,32]. However, the administration of antimicrobial drugs for the treatment of lameness in cattle can be implemented in the case of: (1) interdigital necrobacillosis, (2) ascending infection, (3) severe claw horn disease, and (4) deep digital sepsis [31]. Antimicrobials that can be used in therapy include, among others, ceftiofur, cefoperazone, cefquinome, danofloxacin, enrofloxacin, marbofloxacin, gamithromycin, tildipirosin, tilmicosin, and tulathromycin [33]. The average prevalence of lameness is 22.8% and ranges from 5.1% (in Sweden) to 45% (in the USA), and within the herd ranges from 0% to 88% [31] and amounts to 72% in European dairy herds [30]. Our study indicated that the frequency of lameness depended on the management system. The highest incidence of laminitis was recorded in large dairy herds (mean 7.57), while the lowest was recorded in meat herds. However, each case of infection that requires antibiotic therapy was associated with a large amount of antibiotics due to the weight of the adult animal. Allowing cows to graze on pastures had a positive impact on hoof health, while there was considerable variation in large farms (4.70–18.43). Most of the antibiotics were used to treat foot rot and digital dermatitis. In large beef farms, environmental aetiology diseases dominated, with the largest amounts of antibiotics used for the treatment of respiratory and gastrointestinal diseases (mainly in calves), as well as lameness. Canadian studies indicate that the prevalence of lameness is varied from 19 to 72% [34]. In our own studies, the average for large dairy herds did not exceed 20%.

Bovine respiratory disease (BRD) is the most debilitating health problem in the beef industry. The incidence of the disease in animals is 70–80%, while the mortality rate ranges from 40% to 50% in feedlots in the United States [35,36]. The global economic impact of this disease is expected to exceed USD 3 billion annually [36,37,38]. Today, BRD continues to contribute to significant losses in feedlot performance, health, and carcass quality [35,39,40] and generates annual costs for cow–calf producers of approximately USD 165 million [41]. Negative health and performance are related to: (1) increased mortality, premature culling, and age at first calving, and (2) reduced average daily gains, and milk production in the first lactation [42]. The main causes of excessive levels of BRD in young cattle are related to environmental factors [43]. BRD is difficult to control and prevent as it is a multifactorial disease [37,38] that develops as a result of interactions between various viruses (e.g., bovine respiratory syncytial virus, bovine coronavirus, bovine herpesvirus type 1, bovine parainfluenza virus type 3, bovine viral diarrhoea virus) [39,40,41], bacteria (e.g., *Histophilus somni*, *Mannheimia haemolytica*, *Mycoplasma bovis*, *Pasteurella multocida*) [35,37,40,44] along with factors associated with the host and management practices [40]. In Poland, the prevalence of pathogens causing BRD was the highest for: *Pasteurella multocida*, *Mannheimia haemolytica*, *Bovine coronavirus* (BcoV), *Mycoplasma bovis*, *Histophilus somni*, BPIV-3, and BRSV [39]. Administration of antimicrobials is crucial for effective treatment of diseases. In the USA, a variety of new products have been approved in therapy in BRD, namely: ampicillin trihydrate, ceftiofur crystalline free acid, ceftiofur sodium, ceftiofur hydrochloride, danofloxacin, enrofloxacin, florfenicol, gamithromycin, oxytetracycline, oxytetracycline, penicillin, spectinomycin, tilmicosin, tylosin, tulathromycin, and tildipirosin [37,38]. However, in European countries, such as Germany, the following classes of antibiotics are allowed to be used: aminoglycosides, β-lactams, fluoroquinolones, lincosamides, macrolides, phenicols, tetracyclines, and trimethoprim–sulphonamides [40]. In our study, prevalence of respiratory diseases was diversified among the type of farm, for instance: reaching the highest frequency in large dairy farms at 22.21%, while the mean frequency was much lower in small dairy herds and on meat farms (4.51 and 4.23%, respectively). The data collected indicated that, in a given quarter on large dairy farms, individual calves were treated with antibiotics up to twice, which affected antibiotic consumption, defined as general treatment.

Gastrointestinal diseases (GIDs) are one of the most common disorders in dairy calves before weaning. It is estimated that about 21% of young dairy animals in the United States suffer from GIDs, and 76% of them are treated with antimicrobials, mainly due to diarrhoea [45]. In our case, prevalence of gastrointestinal diseases in calves was the highest in large dairy farms (26.07%), while the lowest in large herds of beef cattle: (2.675). Neonatal calf diarrhoea (NCD) is the leading cause of growth disorder and mortality (at 15–56%) of newborn calves, resulting in economic losses in animal husbandry [46,47,48,49,50]. NCD leads to dehydration, electrolyte imbalances, and metabolic acidosis in animals, and, if no appropriate treatment is implemented, it can be fatal. Long-term consequences include: (1) reduced weight gain, development, and milk production in the first lactation, as well as (2) increased time to the first calving [48]. The causes of NCD are complex and multifactorial, and many factors contribute to its development. The number of pathogenic and infectious agents, such as bacteria (e.g., *Escherichia coli*, *Salmonella enterica*, *Clostridium perfringens*), viruses (rotavirus, bovine coronavirus, bovine astrovirus, bovine viral diarrhoea virus), and protozoa (e.g., *Cryptosporidium* spp., *Giardia duodenalis*, *Eimeria* spp.) are the main factors causing diarrhoea [47,48,50]. Therefore, *E. coli* constitutes the most crucial bacterial agent, and due to its increasing resistance to antibiotics, treatment is complicated and still results in many deaths [49]. On the other hand, non-infectious agents include nutritional, immunological, environmental, and management factors [48] and, in addition, veterinary treatment [51]. Diarrhoea is responsible for 57% of weaned calf mortality, and 20% of calf mortality can result in a 38% decrease in net income. This condition can decrease animal growth and reproductive performance, as well as milk production in the advanced stage of lactation [50]. Antimicrobial therapy for NCD is still a common practice in animal husbandry [50,52]. Data collected from four European countries showed that almost 53% of farmers and veterinarians administered antimicrobials to newborn calves with diarrhoea [52]. In our study, only those morbidities that were associated with antibiotic administration were included, according to the established methodology. In the United States, the Food and Drug Administration approved the use of the following agents for the treatment of gastrointestinal diseases in calves: ampicillin, amoxicillin, chlortetracycline, oxytetracycline, sulphamethazine, and tetracycline [45]. 

The transparency of the methods used to calculate indicators is crucial to ensuring comparisons of use between different livestock populations [53]. Electronic systems enable significant progress in the monitoring of antibiotic use [54]. The Regulation (EU) 2019/6 of the European Parliament and the Council of 11 December 2018 on veterinary medicinal products and repealing Directive 2001/82/EC sets out new rules for the use of antimicrobial agents (antibiotics) in veterinary medicine, which affects the use of medications in animals to prevent the rise of bacterial resistance [16]. In Poland, with the implementation of these regulations, national antibiotic consumption records systems will soon be implemented. Today, an increasing number of countries are implementing efforts to reduce the use of antibiotics in food-producing animals. In 2021, sales (in tonnes of active substance of antibiotic veterinary medicinal products) in the 31 European countries ranged from 0.5 (in Iceland) to 1296.5 mg·PCU^−1^ (in Spain). Countries with low sales also include Luxembourg (1.5 mg·PCU^−1^), Malta (1.6 mg·PCU^−1^), Latvia (3.9 mg·PCU^−1^), Estonia (5.3 mg·PCU^−1^), and Norway (5.5 mg·PCU^−1^). In contrast, the highest sales were recorded in the following: Poland (775.1 mg·PCU^−1^), Italy (661.7 mg·PCU^−1^), Germany (590.7 mg·PCU^−1^), and France (349.3 mg·PCU^−1^). Oral solutions were the highest selling product form, accounting for 57.9% of the total sales (mg·PCU^−1^), followed by premixes (21.8%), injectable products (12.6%), oral powders (6.6%), intramammary products (0.71%); the remaining sales (0.42%) corresponded to oral pastes, boluses, and intrauterine products [55]. Considering Sweden, as a country that administers antibiotics at very low levels, it is worth mentioning that the incidence of systematic antibiotic treatment per 100 cow-years decreased by 50% between 2001 and 2017, while the productivity of Swedish dairy cows is among the highest in Europe [56]. This proves that it is possible to ensure a sufficient amount of safe food, free from antibiotics, in a fully sustainable way. The monitored antibiotic use in the UK indicates a lower average consumption of antibiotics in mg·PCU^−1^ compared to our own studies [57]. The authors of these studies suggest that the stock of drugs available to farmers (stored on the farm) likely influences treatment decisions. The large herds included in our studies have an increased frequency of veterinarian visits, which in practice means initiating treatment. At the same time, veterinarians should consider rational selection of antibiotics, taking into account the reduction in the use of restrict antibiotics for dry cow therapy. Danish studies indicate that the reduction of antibiotic resistance is sometimes based on the personal experience of veterinarians rather than scientific evidence. Furthermore, it was noted that some veterinarians do not view the risk of antibiotic resistance as a daily threat related to their work [58]. More research in this area is needed to provide a robust information base for responsible future decision-making on the use of medications in dairy farming.

## 5. Conclusions

Determining the causes of high levels of antibiotic usage in cattle farms is possible through accurate monitoring, detailed evaluation of the groups of antibiotics used, and assessment of the frequency of illnesses, as well as their causes. Large dairy cattle farms are characterised by the highest use of antibiotics, which is due to the high frequency of illnesses, and consequently, the treatment of calf diseases (diarrhoea, lung inflammations) and cow diseases (general treatment and mastitis). Cattle on large beef farms suffer mainly from diseases of environmental aetiology. The evaluation of antibiotic resistance was carried out on a very limited scale before treatment began. The use of restrict antibiotics in some cases was unjustified (antibiotics for dry cow therapy). Given the use of antibiotics in cattle herds, holistic reduction efforts are needed in the future, with incentives and motivation for breeders to focus on disease prevention and better management of animal health. Future research should focus on developing appropriate local studies on antimicrobial resistance and possible approaches to reduce the use of antimicrobial agents, along with reduction strategies that have real-world applications in cattle breeding.

## Figures and Tables

**Table 1:** Type of herd, production, health, and characteristics of the cattle farms studied.

Characteristics	Medium Dairy Cow Farms, Means (Range)	Large Dairy Cow Farms, Means (Range)	Large Herds of Beef Cattle, Means (Range)
Total herd size	86.76	1498.43	928.42
Number of cows in milk	32.49	514.00	345.38 ^2^
Number of calves	31.33	506.06	339.94
Milk yield	7205 (6345–8102)	11,455 (10,235–12,670)	–
Dry cow therapy (DC)	29.34 (8.35–56.98)	31.72 (14.56–65.23)	–
Microbiology ^3^	0.34 (0.00–20.23)	2.43 (1.02–6.23)	–
Disease prevalence (for which antibiotics were used)			
Mastitis ^1^	5.98 (0.29–27.20)	15.85 (6.70–36.65)	0.02 (0.0–0.2)
Lameness ^1^	1.25 (0.0–6.75)	7.57 (4.70–18.43)	0.84 (0.60–4.30)
Gastrointestinal diseases in calves ^1^	6.09 (0.55–40.14)	26.07 (9.50–50.87)	2.67 (0.65–35.14)
Respiratory diseases in calves ^1^	4.51 (0.25–42.56)	22.21 (2.50–87.52)	4.23 (0.29–34.72)
Respiratory diseases in cows ^1^	0.62 (0.0–2.43)	0.85 (0.0–2.14)	0.01 (0.00–0.12)
Other *	1.68 (0.95–6.82)	(3.50–18.90)	0.23 (0.14–0.71)

^1^ Average per year; ^2^ Number of beef cows; ^3^ Microbiological tests were performed before using antibiotics * inflammation of the navel, *metritis*, displacement of abomasum, etc.

**Table 2:** Antibiotic usage in the studied farms was evaluated based on the production type (mg·PCU^−1^).

Characteristics	Medium Dairy Cow Farms, Means (Range)	Large Dairy Cow Farms, Means (Range)	Large Herds of Beef Cattle, Means (Range)	SEM
Total antimicrobials usage	5.14 ^A^	18.29 ^B^	4.84 ^A^	1.33
General treatment	4.30 ^A^	14.22 ^B^	4.83 ^A^	1.02
LC	0.41 ^A^	3.39 ^B^	–	0.25
DC	0.43 ^A^	0.68 ^B^	–	0.06
Overall usage broken down by group				
Group B	1.21 ^A^	6.41 ^B^	1.02 ^A^	1.37
Group C	2.37 ^aA^	3.35 ^B^	1.66 ^bA^	0.29
Group D	1.56 ^aA^	8.53 ^B^	2.16 ^bA^	1.35
Antibiotic usage in general therapy				
Group B	1.03 ^A^	5.05 ^B^	1.02 ^A^	1.03
Group C	1.97 ^A^	2.86 ^b^	1.66 ^A^	0.46
Group D	1.30 ^aA^	6.31 ^B^	2.16 ^bA^	0.87
Antibiotic usage in lactating cow therapy (LCT)				
Group B	0.02 ^A^	0.80 ^B^	–	0.33
Group C	0.34 ^a^	0.46 ^b^	–	0.26
Group D	0.05 ^a^	2.14 ^b^	–	0.37
Antibiotic usage in dry cow therapy (DCT)				
Group B	0.16 ^A^	0.56 ^A^	–	0.39
Group C	0.06	0.03	–	0.08
Group D	0.21 ^A^	0.09 ^B^	–	0.56

A,B—means in rows marked with different lowercase superscripts differ significantly at *p* < 0.01; a,b—means in rows marked with different lowercase superscripts differ significantly at *p* < 0.05; SEM—standard error of mean.

## Data Availability

The data presented in this study are available upon request from the corresponding author.

## Supplementary Materials

The following supporting information can be downloaded at: https://www.mdpi.com/article/10.3390/ani14131889/s1, Table S1: General information about the herd (regardless of the direction of use); Table S2: Detailed sheet to record each case of antibiotic treatment excluding mammary gland disorders; Table S3: Detailed sheet for recording *mastitis* treatment in lactation; Table S4: Detailed worksheet for recording antibiotic use during drying out of cows.
