# Supplementary material for: Comparison of Antibiotic Use and the Frequency of Diseases Depending on the Size of Herd and the Type of Cattle Breeding
Source: Animals (Basel). 2024 Jun 27;14(13):1889. doi: 10.3390/ani14131889 (PMC11240473; doi:10.3390/ani14131889)
Supplement: Supplementary file 1 [file animals-14-01889-s001.zip › animals-3015386-supplementary.pdf]

Supplementary materials

The following Supplementary materials present the individual pages of the spreadsheet used to collect data from each quarter of the year on the farm (Excel file). The original file contains a database with antibiotics available on the Polish market and formulas for converting the numerical data.

Table S1. General information about the herd (regardless of the direction of use).

General information				
Full name				
Herd location number				
Start date				
End date				
**Number of cattle in the herd**				
Item	End of previous period	End of current period	Mean	Recalculation of body weight according to EMA
Cows				
Heifers				
Calves (under 6 months)				
Bulls				
**Other information**				
Milk yield				
Breed				
Other				

Table S2. Detailed sheet to record each case of antibiotic treatment excluding mammary gland disorders.

General treatment																
Date	Cattle category	Number of animals treated	Diagnosis	Antibiotic (trade name)	Route of administration	Daily dose	Number of injections/ number of treated animals	Diagnosis	Amount used	Unit	Restrict (0 = no, 1 = yes)	Critically important, mg/PCU	Antibiotic use, mg/PCU	EMA class (A-D)	Antibiogram (0 = no, 1 = yes)

Table S3. Detailed sheet for recording *mastitis* treatment in lactation.

Mastitis treatment									
Date	Route of administration	Antibiotic (trade name)	Antibiogram (0 = no, 1 = yes)	Number of turbo syringes (throughout the treatment period; number of administrations; amount used)	Unit	Restrict (0 = no, 1 = yes)	Critically important, mg/PCU	Antibiotic use, mg/PCU	EMA class (A-D)

Table S4. Detailed worksheet for recording antibiotic use during drying out of cows.

Dry cows treatment								
Date	Route of administration	Antibiotic (trade name)	Number of turbo syringes (throughout the treatment period; amount used)	Unit	Critical (0 = no, 1 = yes)	Critically important, mg/PCU	Antibiotic use, mg/PCU	EMA class (A-D)
